# Genome-wide analysis of WD40 protein family and functional characterization of *BvWD40-82* in sugar beet

**DOI:** 10.3389/fpls.2023.1185440

**Published:** 2023-06-02

**Authors:** Zhirui Wu, Tingyue Zhang, Jinna Li, Sixue Chen, Inga R. Grin, Dmitry O. Zharkov, Bing Yu, Haiying Li

**Affiliations:** ^1^ Engineering Research Center of Agricultural Microbiology Technology, Ministry of Education & Heilongjiang Provincial Key Laboratory of Plant Genetic Engineering and Biological Fermentation Engineering for Cold Region & Key Laboratory of Molecular Biology, College of Heilongjiang Province & School of Life Sciences, Heilongjiang University, Harbin, China; ^2^ Department of Biology, University of Mississippi, Oxford, MS, United States; ^3^ Department of Natural Sciences, Novosibirsk State University, Novosibirsk, Russia; ^4^ Institute of Chemical Biology and Fundamental Medicine, Siberian Branch of the Russian Academy of Sciences, Novosibirsk, Russia

**Keywords:** sugar beet (*Beta vulgaris* L.), WD40 proteins, expression profile, salt stress, *BvWD40-82*, function

## Abstract

Sugar beet is one of the most important sugar crops in the world. It contributes greatly to the global sugar production, but salt stress negatively affects the crop yield. WD40 proteins play important roles in plant growth and response to abiotic stresses through their involvement in a variety of biological processes, such as signal transduction, histone modification, ubiquitination, and RNA processing. The WD40 protein family has been well-studied in *Arabidopsis thaliana*, rice and other plants, but the systematic analysis of the sugar beet WD40 proteins has not been reported. In this study, a total of 177 *Bv*WD40 proteins were identified from the sugar beet genome, and their evolutionary characteristics, protein structure, gene structure, protein interaction network and gene ontology were systematically analyzed to understand their evolution and function. Meanwhile, the expression patterns of *BvWD40s* under salt stress were characterized, and a *BvWD40-82* gene was hypothesized as a salt-tolerant candidate gene. Its function was further characterized using molecular and genetic methods. The result showed that *BvWD40-82* enhanced salt stress tolerance in transgenic *Arabidopsis* seedlings by increasing the contents of osmolytes and antioxidant enzyme activities, maintaining intracellular ion homeostasis and increasing the expression of genes related to SOS and ABA pathways. The result has laid a foundation for further mechanistic study of the *BvWD40* genes in sugar beet tolerance to salt stress, and it may inform biotechnological applications in improving crop stress resilience.

## Introduction

WD40 proteins are evolutionarily conserved and widely distributed in eukaryotic organisms. They tend to consist of 4 to 16 WD40 domains, which are also known as the WD40 repeats (WDR) (Smith et al., 1999). The WDR is generally composed of 40 to 60 amino acid residues, with a GH (glycine-histidine) dipeptide at the N-terminus and WD (tryptophan-aspartate) dipeptide at the C-terminus (Neer et al., 1994). The WDR typically folds into a highly stable seven-bladed β-propeller (Stirnimann et al., 2010), connected by an N-terminal amino acid residue in a closed loop, which determines specific protein functions (Mishra et al., 2012).

Members of the WD40 protein family have been identified in many plants. For example, the reference plant *Arabidopsis* has 230 WD40 proteins (Li et al., 2014). Among the cash crops reported, there were 225 WD40 proteins in red sage (*Salvia miltiorrhiza*) (Liu et al., 2020), 187 WD40 proteins in Rosaceae (*Rosa chinensis* ‘old blush’) (Sun et al., 2020), 42 WD40 proteins in walnut (*Juglans regia*), and 204 WD40s in fig (*Ficus carica*) (Chen et al., 2022a; Fan et al., 2022). Furthermore, among the food crops, wheat (*Triticum aestivum*) has 743 WD40 proteins (Hu et al., 2018), potato (*Solanum tuberosum*) has 168 WD40 proteins (Tao et al., 2019), and rice (*Oryza sativa*) has 200 WD40 proteins (Ouyang et al., 2012). However, the WD40 protein family in sugar beet (*Beta vulgaris* L) has not been reported. As one of the largest protein families, WD40 proteins were once regarded as scaffolds for recruiting other molecules to form functional complexes or participate in protein-protein interactions (Li and Roberts, 2001). In recent years, a large number of studies have shown that WD40 proteins have a variety of biological functions. In animals, they are involved in many biological processes, including signal transduction (Liang et al., 2022), histone modification (Lorton et al., 2020), DNA damage response (Choi et al., 2022), transcriptional regulation (Mo et al., 2023), ribosome biosynthesis (Barandun et al., 2018), protein degradation, and apoptosis (An et al., 2022; Cai et al., 2022). In plants, WD40 protein is generally considered to be an important regulator of several biological processes, e.g., anthocyanin biosynthesis (Ji et al., 2023), flowering meristem development (Park et al., 2019), gametogenesis (Shi et al., 2005), embryogenesis (Kim et al., 2021), and yield (Chen et al., 2022b). In addition, genes encoding WD40 proteins also play important roles in plant response to abiotic stresses. For instance, overexpression of a *TaPUB1* gene in tobacco enhanced tobacco salt tolerance by reducing Na^+^ accumulation and reactive oxygen species (ROS) in the transgenic plants and increasing the expression of antioxidant-related genes (Zhang et al., 2017). In addition, an *AtXIW1* gene in *Arabidopsis* plays a positive role in ABA response. Mutation of *AtXIW1* inhibited the induction of ABA-responsive genes and the accumulation of *ABI5*, and led to rapid proteasome degradation of the ABI5 (Xu et al., 2019). Furthermore, inhibition of a rice *OsRACK1A* expression enhanced the rice salt tolerance through maintaining high K^+^/Na^+^ and reducing the accumulation of malondialdehyde (MDA). *Os*RACK1A was found to interact with many salt stress response proteins to decrease the salt tolerance of rice (Zhang et al., 2018). Recently, overexpression of another *TaWD40-4B.1* gene increase the biomass of transgenic wheat under drought stress. The *Ta*WD40-4B.1 protein interacts with *Ta*CAT3 protein to promote their oligomerization and catalase activity under drought stress, leading to improved drought tolerance of the transgenic wheat (Tian et al., 2023).

Sugar beet is an *Amaranthaceae* biennial herbaceous plant, one of the world’s important sugar crops, accounting for 20%-25% of the world’s annual sugar production (Khan et al., 2019). Sugar beet is also an important cash crop in northeast China, and its root has high economic value (Thiruvengadam et al., 2022). Sugar beet is also a halophyte tolerant to salt and alkali stresses. With the increase of saline-alkali land in the world, crop production and food security have become a grand challenge (Kopecká et al., 2023). Systematic identification of plant salt tolerance genes toward improving crop stress resilience and increasing yield is urgently needed (Ma et al., 2017; Ji et al., 2019). Since the WD40 proteins are involved in many biological processes including plant growth and stress response, we hypothesize that some WD40 protein encoding genes in halophyte sugar beet play important roles in plant salt stress tolerance. In this study, we analyzed the sequences of the WD40 family proteins in the sugar beet genome. The expression profiles of the *BvWD40* genes under salt stress were characterized. One of the genes, *BvWD40-82*, was found to confer plant salt stress tolerance. The result not only highlights the utility of gene functional analysis informed by genome-wide informatics, but also provides important resources for the community to further explore the roles of *WD40* genes in crop stress resilience and yield.

## Materials and methods

### Identification of *Bv*WD40 proteins in sugar beet

The seed file for the WD40 domain was downloaded from the InterPro database (www.ebi.ac.uk/interpro/entry/pfam/PF00400/). A Hidden Markov Model (HMM) of WD40 domain was constructed using the HMMER program (Mistry et al., 2013), and the NCBI protein database of sugar beet (www.ncbi.nlm.nih.gov/genome) was searched and compared. *E*-value was used to screen candidate proteins (*E*-Value<0.05) and SMART (http://smart.embl-heidelberg.de/) was used to verify and confirm that all the *Bv*WD40 members contain the WD40 domain. The conserved motifs of *Bv*WD40s were predicted by the MEME program (meme-suite.org/tools/meme). The domain and conserved motifs of *Bv*WD40s were visualized by TBtools (Chen et al., 2020). The theoretical molecular weight and isoelectric point of each *Bv*WD40 were analyzed by using the Expasy tool (web.expasy.org/compute_pi/).

### Phylogenetic analysis

The sequences of 177 *Bv*WD40 proteins were downloaded from the NCBI, and then multi-sequence alignment of the *Bv*WD40s was performed by the Clustal W (Larkin et al., 2007). The trimAl was used to trim the sequences (Capella-Gutiérrez et al., 2009). The parameter was set as the fraction of sequences with allowed gaps of 0.8, the minimum average similarity of 0.001, and the minimum percentage of positions retained in the original route of 80 percent. The pruned files were imported into IQ-TREE 2 (Minh et al., 2020), and the phylogenetic tree was established by the maximum likelihood method with 1000 replicates of bootstrapping.

### Gene structure, chromosomal location, and gene duplication analysis of the *BvWD40* genes

The genome annotation file, coding sequence (CDS), and the sequences of *BvWD40* genes were downloaded from NCBI. The gene structure pattern map of *BvWD40s* was obtained from the GSDS website (gsds.gao-lab.org/). We used TBtools to map *BvWD40s* chromosomal location information. Based on the previous data (Li et al., 2014), we supplemented the *Arabidopsis WD40* genes with newly identified *WD40* genes: *AT1G05631.1*, *AT1G51690.1*, *AT1G655801.1*, *AT2G31830.1*, *AT2G439001*, *AT3G56990.1*, and a total of 236 *AtWD40s* were obtained. The collinearity relationship between *BvWD40s* and *AtWD40s* was analyzed by MCScanX (Wang et al., 2012), and the collinearity map of sugar beet and *Arabidopsis* was generated by Circos (Krzywinski et al., 2009).

### Interaction networking, expression profiling, and gene ontology analysis

The *Bv*WD40 proteins interaction network was obtained from the String website (cn.string-db.org/) (Szklarczyk et al., 2015) and visualized using the Cytoscape software (Shannon et al., 2003). The RNA-seq data of *B. vulgaris* were downloaded from the NCBI SRA database (Accession: PRJNA666117). The transcriptome data of the *BvM14* line under salt stress were generated and stored in the Li laboratory. The expression profile of *BvWD40* genes was used by TBtools software. The basic data of gene ontology analysis were obtained from the GO network database (geneontology.org/), and GO annotation and enrichment of the *BvWD40* genes were performed using TBtools and visualized by an online tool (www.bioinformatics.com.cn).

### Plant materials and salt stress treatment

The *BvM14* line is a monomeric additional line obtained by crossing and backcrossing between diploid cultivated sugar beet and tetraploid wild sugar beet, which was created and propagated by the Li lab (*Beta vulgaris* L., VV+1C, 2*n*=18 + 1) (Li et al., 2022b). The seeds were disinfected and cultured in a hydroponic system as previously reported (Ma et al., 2017; Ji et al., 2019). After growing to the third pair of fully expanded leaves, leaves and roots were sampled to extract total RNA using the TRIzol method (Meng and Feldman, 2010). Three biological replicates were conducted. Primer 3 plus (www.primer3plus.com) was used to design specific primers, and *18sRNA* was used as the reference. qRT-PCR was performed using SYBR, and the relative gene expression was calculated using the 2^-ΔΔCt^ method (Schmittgen and Livak, 2008).

*A. thaliana* Columbia ecotype (Col-0) seeds were obtained from the ABRC (abrc.org), and were germinated in 1/2 MS medium under 300 µmol/m^2^ s light intensity, 14 h light and 10 h darkness. After eight days, the seedlings were transferred to a new MS medium (with or without 150 mM NaCl) for 7 days to further screen transgenic plants, or observe their growth phenotypes under salt stress. After soil and vermiculite were mixed at 2:1, and sterilized at 180 °C in a dryer, the seeds were sowed in the mixed soil. After 21 days of culture (relative humidity 65%-75%, 22 °C, light 16 h/dark 8 h), the seedings were treated with 150 mM NaCl salt stress, and three plants with consistent growth were selected from each line for subsequent physiological/biochemical analyses and RNA extraction.

### Subcellular localization of the *BvWD40-82* and generation of transgenetic lines

The *BvWD40-82* gene was constructed in the pCAMVBIA2300-35S-eYFP vector and its subcellular localization was observed by laser scanning confocal microscopy (FV1200, Olympus). The nucleotide sequence of *AtUTP18*, the homologous gene of the *BvWD40-82* gene in *Arabidopsis*, was obtained by NCBI online BLASTN software. In order to construct *utp18* mutation plants to characterize the function of this gene, the online tool CRISPR-P2.0 (cbi.hzau.edu.cn/cgi-bin/CRISPR2/CRISPR) was used to select appropriate targets and construct CRISPR/Cas9 vectors (pNGG2F vector). After infecting the flowers of wild-type *Arabidopsis*, the *utp18* homozygous mutant line was obtained by Hygromycin B (30 mg/L) screening and TA clone sequencing. The *BvWD40-82* gene was constructed into the pCAMBIA1300-35S-3xFLAG vector, the wild-type *Arabidopsis* and *utp18* mutant lines were transformed by floral infestation, and the seeds of each plant were collected and placed in MS medium which contains Hygromycin B (30 mg/L), and the seedlings with normal growth were placed in the soil for culture after about ten days. The DNA and RNA from the leaves of each transgenic plant were extracted after four weeks for DNA verification and RT-PCR. The T3 generation homozygous transgenic lines (Heterologous overexpression, OE#16, OE#17, OE#18; Heterologous complementation, CO#1, CO#2, CO#5) were used for further analysis.

### Physiological and biochemical index determination

Root length of each transgenic seedling and wild-type seedling with or without 150 mM NaCl treatment in the MS medium was determined. The dry weight and fresh weight (with or without 150 mM NaCl in the soil) were also analyzed. The MDA content, SOD and POD enzyme activities were analyzed using previously published methods (Ma et al., 2017; Ji et al., 2019); Betaine content was determined at 525 nm (Swarna et al., 2013); Na^+^, K^+^, and Ca^2+^ contents were analyzed by a flame atomic absorption method, as previously described (Gao et al., 2016).

## Results

### Identification of WD40 proteins in sugar beet

A total of 177 *Bv*WD40s were obtained by removing redundant proteins and repetitive sequences. Based on the location of the corresponding genes of these proteins on the nine chromosomes of the sugar beet, they were named *BvWD40-1* to *BvWD40-177*. In silico analysis showed that the physicochemical properties and sequence composition of the *Bv*WD40 proteins varied largely, e.g., the molecular weights (MW, 13.79-398.63 kDa) and isoelectric points (pI, 4.25-9.67) spanned a wide range. The sequence length of *Bv*WD40s ranges from 126 to 3599 amino acids, with an average length of 627.7 amino acids (**Supplementary Table 1**). SMART indicated that the *Bv*WD40s contained 1 to 13 WD repeats.

### Conservative domain and motif analysis of *Bv*WD40s

The results of protein structure analysis showed that all the *Bv*WD40 proteins contained the WD40 domain, and more than 35 additional functional domains in total (**Supplementary Table 2**). Most (49) *Bv*WD40s had six WD40 repeats, followed by seven WD40 repeats in 43 *Bv*WD40s. Thirty-six *Bv*WD40s had their WD40 domains at the C-terminus of their other domains. Except for the WD40 domain, the frequency of the LisH domain was the highest (12 *Bv*WD40s). Studies have shown that the LisH domain affects rice growth and reproduction (Gao et al., 2012), suggesting that *Bv*WD40 proteins with the LisH domain may also be involved in similar processes. Various other domains were present but infrequently, such as the ATG16 domain only in *Bv*WD40-106 and the BCAS3 domain only in *Bv*WD40-57 and *Bv*WD40-64. These two domains are involved in cell autophagy of yeast (Xiong et al., 2018; Yamada and Schaap, 2021). The UTP15 domain (only in *Bv*WD40-20), was a component of the UtpA complex involved in the assembly of small ribosomal subunits (Kornprobst et al., 2016). However, the *Arabidopsis AtMSI4* gene encoding a WD40 protein contains six WD40 domains and a CAF1C domain, and may be involved in nucleosome assembly, but some studies have shown that *AtMSI4* is involved in regulation of flowering in *Arabidopsis* (Pazhouhandeh et al., 2011). As another example, the RNAi-*AtATG18a* plants are more sensitive to salt and mannitol and defective in autophagosome formation in *Arabidopsis*, it indicated that *AtATG18a* may plays an important role in plant autophagy and abiotic stress. It should be noted that the *At*ATG18a contains only the WD40 domain (Liu et al., 2009).

We predicted 50 conserved motifs for 177 *Bv*WD40s using the MEME tool (**Supplementary Figure 1**), among which the most highly conserved motifs were Motif 1 (173 times), Motif 2 (171 times) and Motif 4 (171 times). The frequencies of tryptophan (W) and aspartate (D) were the highest in Motif 1 and Motif 2, and the frequencies of glutamate (G) and histidine (H) were the highest in Motif 4, the results also showed that the amino acid composition of the WD40 domain was quite different. Interestingly, some motifs occur less frequently, such as Motif 6 (8 times) and Motif 18 (61 times), which have the WDxR motif (**Supplementary Figure 2**). The WDxR motifs are often found in DCAFs, which have been proposed as substrate recruiting proteins for the E3 ubiquitin ligase complex Cullin4-DDB1 (Mistry et al., 2020). In addition, previous studies have shown that the WDxR motif of the DCAF WD40 domain in plants is a critical motif for interacting with the DDB1 protein. It may also be involved in a variety of plant developmental pathways (Zhang et al., 2008).

### Subfamily classification and phylogenetic analysis

**Figure 1:**
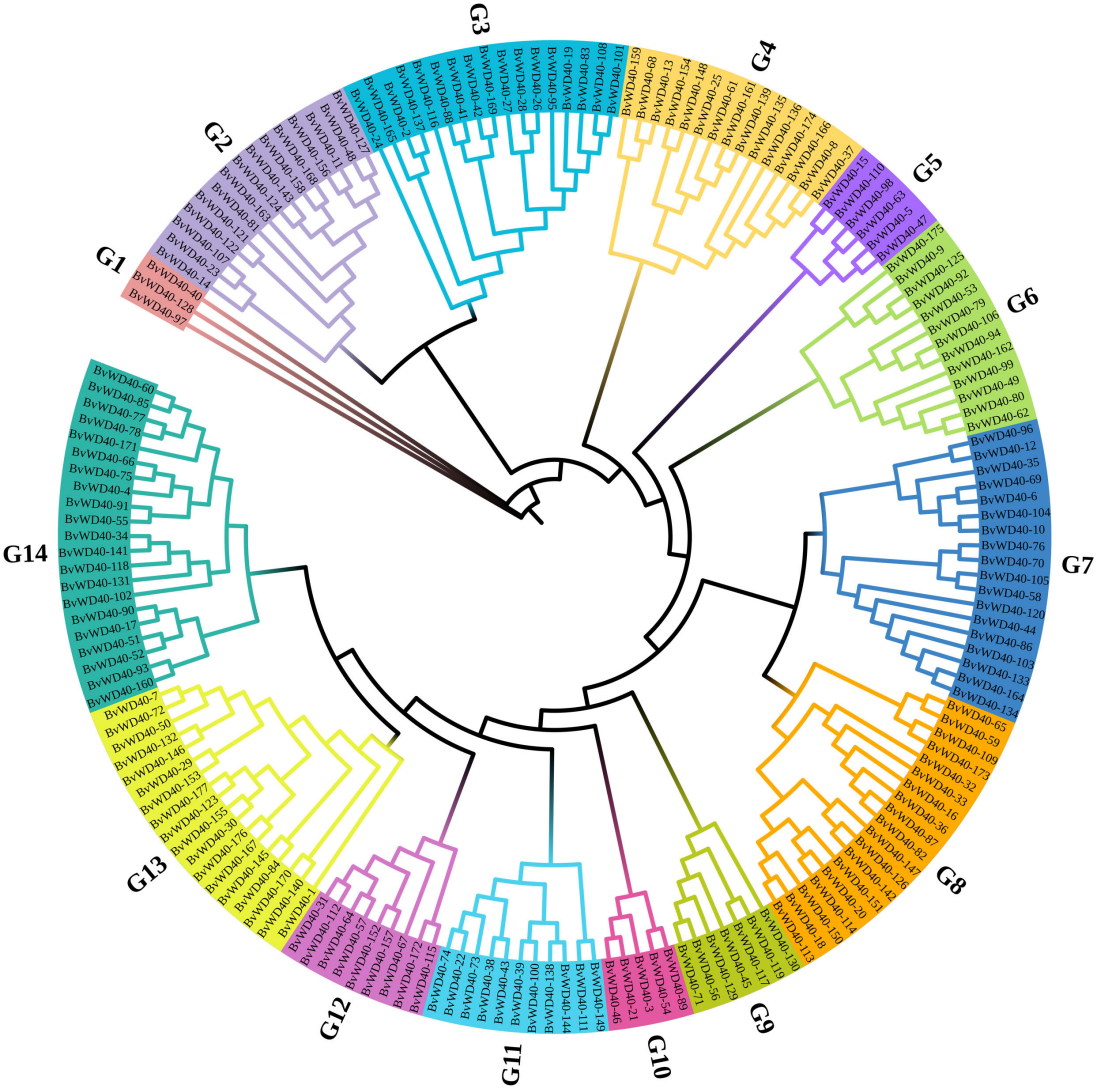
Phylogenetic tree of the WD40 proteins from sugar beet. The phylogenetic tree was constructed using ClustalW, trimAl, and IQ-Tree software with the maximum likelihood method and 1000 bootstrap replicates. Branch lines in different colors represented different groups (G1 to G14).

We divided the *Bv*WD40 proteins into 13 subfamilies based on their domain composition and the roles of different domains in biological processes (**Table 1**). Subfamily A is the largest in sugar beet with 108 members. It contains only the WD40 domain. Subfamily I is the second largest family with 16 members that contain LisH and IPPc domains associated with plant growth and development. Other subfamilies also have functional domains that play different key roles, such as ribosome synthesis, ubiquitination, nucleosome assembly, and vesicle transport, suggesting that *Bv*WD40s may be master regulators in various processes. The *Bv*WD40 proteins were divided into 14 different clusters (G1-G14) based on their sequence homology, among which G1 has only 3 members and was the smallest cluster and G14 was the largest cluster with 21 members (**Figure 1**). The structure similarity of the members in each cluster is high, and the composition and order of the conserved domain are consistent (**Supplementary Figure 1**). It is worth noting that the clusters are not the same as subfamilies, possibly due to the large differences in amino acid composition of domains that maintain similar functions.

**Table 1:** Domain composition, function and size of the 13 subfamilies of 177 *Bv*WD40s.

Subfamily	Domain	Role	Number	Reference
Subfamily A	only WD40 domain	Development, Abiotic	108	(Xu et al., 2019)
Subfamily B	UTP15_C/UTP12/UTP13/UTP21/Sof1/NLE/BOP1NT domain	Ribosome Biogenesis	10	(Barandun et al., 2018)
Subfamily C	RING/UBOX/FBOX/Znf_C3H1/Znf_C2H2/ANAPC4/PFU and PUL domain	Ubiquitination	12	(Stone et al., 2005)
Subfamily D	Hira/CAF1C_H4-bd domain	Nucleosome Assembly	6	(Tripathi et al., 2015)
Subfamily E	DENN/Sec_16/Coatomer_WDAD/BEACH domain	Vesicular Transport	10	(Steffens et al., 2017)
Subfamily F	BCAS3/ATG16 domain	Autophagy	3	(Yamada and Schaap, 2021)
Subfamily G	PRP4/Pro_ismorase domain	Protein Processing	2	(Ayadi et al., 1998)
Subfamily H	Katanin_con80 domain	Microtubule-Severing	2	(McNally et al., 2000)
Subfamily I	BROMO/LisH/IPPc/Raptor_N domain	Plant Development	16	(Gao et al., 2012)
Subfamily J	Protein Kinase domain	Protein Kinase	2	(Bheri et al., 2021)
Subfamily K	zf_UDP and Cellulose_synt with Glyco_trans_2_3 domain	Cellulose Synthesis	1	(Song et al., 2019)
Subfamily L	Mcl1_mid domain	Damage Survival	1	(Williams and McIntosh, 2005)
Subfamily M	BING4CT(NUC141)/NUC153/DUF3337/PD40 domain	Others	4	

### Gene structure, chromosomal location, and duplication of *BvWD40* genes

The gene structure of the 177 *BvWD40* genes was analyzed by the GSDS software (**Supplementary Figure 3**). The number of exons in the *BvWD40s* varied greatly. *BvWD40-10* has 39 exons, while 10 *BvWD40s* have only one exon. On average, the *BvWD40s* have 10.8 exons and 9.8 introns.

**Figure 2:**
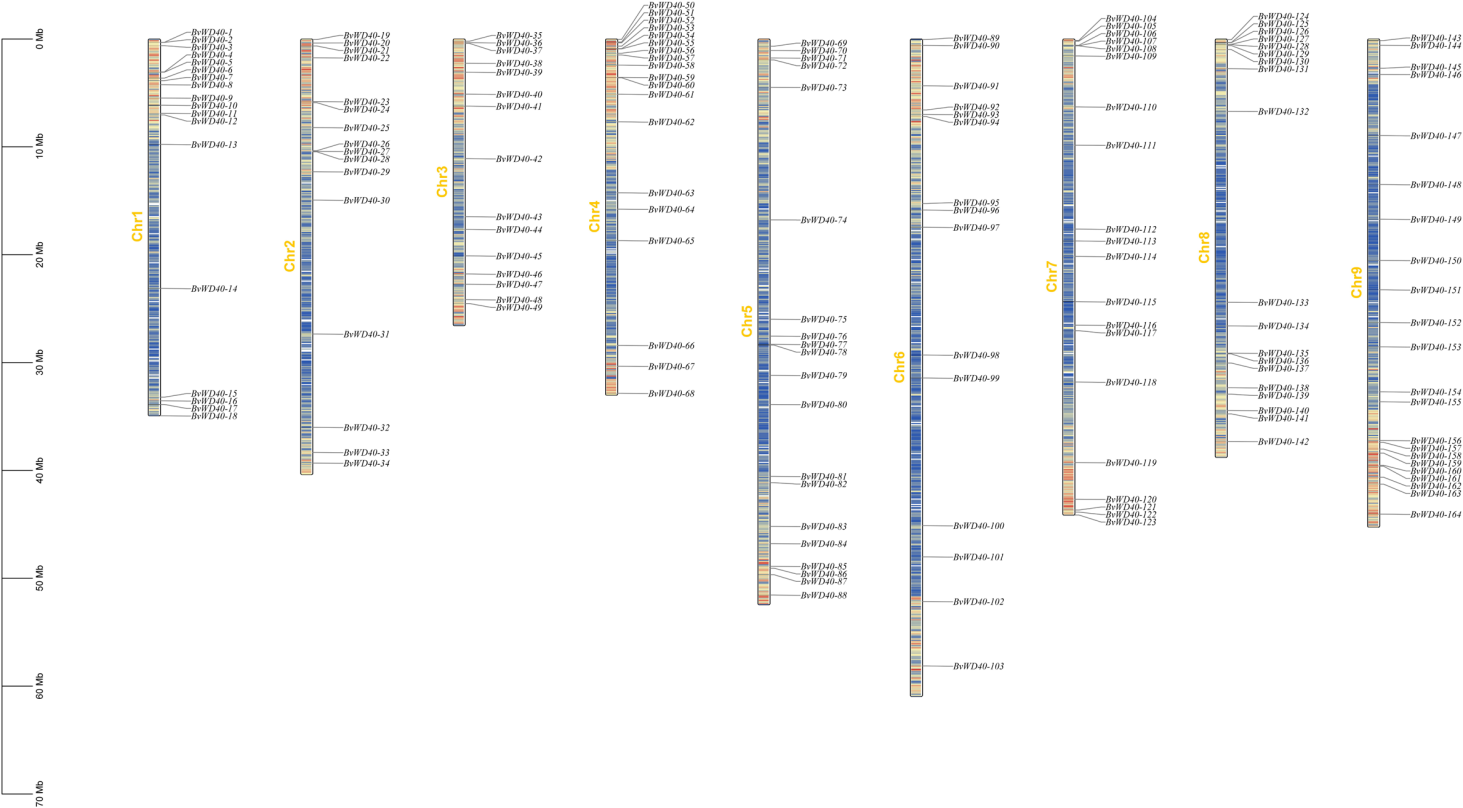
Locations of *BvWD40* genes on the sugar beet chromosome. According to the location information of the *BvWD040s*, *BvWD40s* was marked on each chromosome of sugar beet. There was a number marker on the left side of each chromosome, the length was synchronized with the scale, and the gene density was distributed according to the color (low density: blue, high density: red).

Since 13 *BvWD40s* were not fully assembled, we mapped the remaining 164 *BvWD40s* to the sugar beet chromosomes using positional information. The *BvWD40s* were widely distributed on the nine chromosomes (**Figure 2**). Chromosome 9 has 22, the highest number of *BvWD40s*, while chromosome 3 and chromosome 6 each has 15, the lowest number.

**Figure 3:**
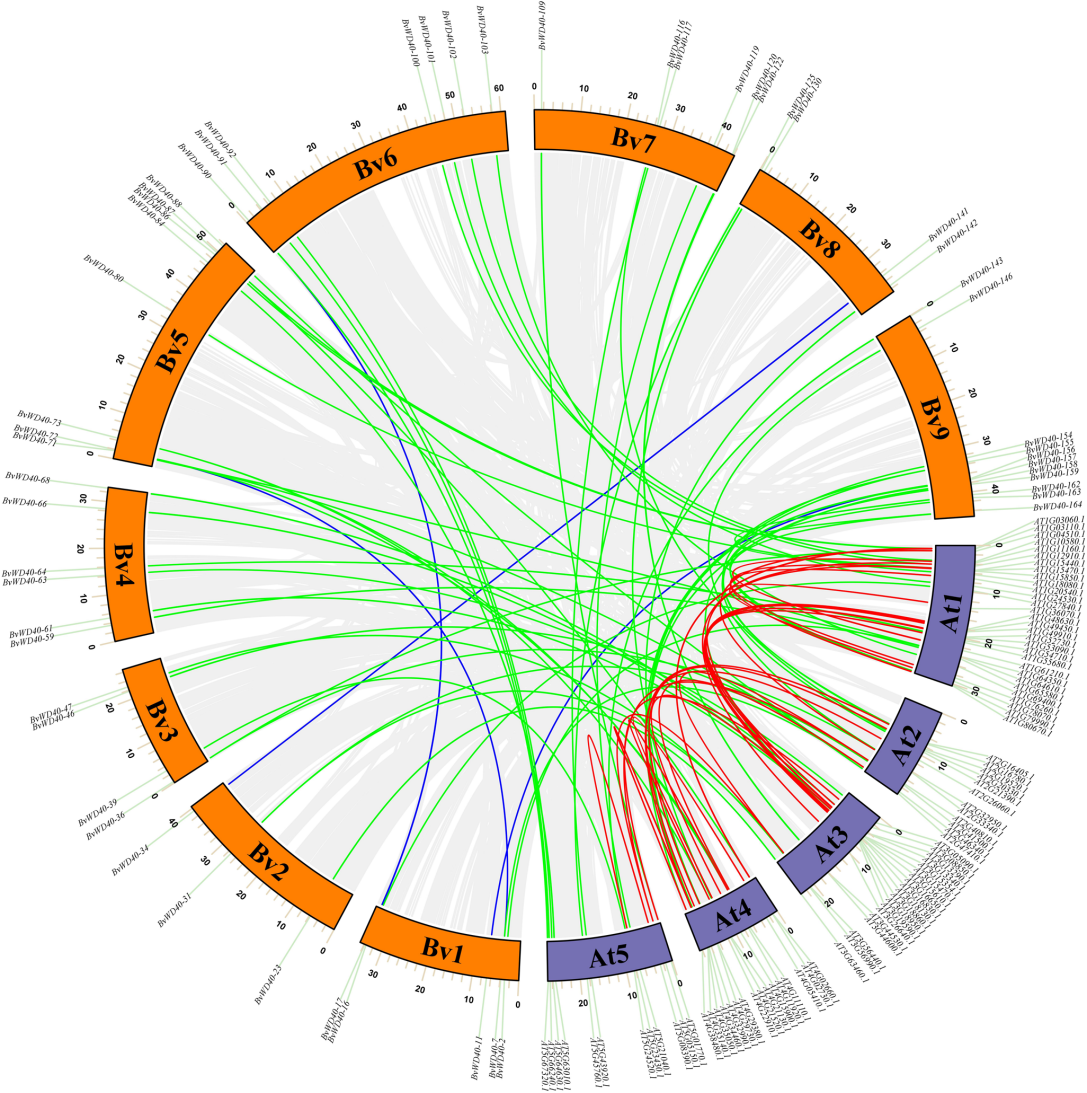
Collinearity analysis of the *WD40* genes in sugar beet and *Arabidopsis*. The sugar beet and *Arabidopsis* chromosomes were indicated by the orange and purple block, respectively. Grey lines indicate all existing genes for a linear relationship, the blue line represents the sugar beet with collinearity of *WD40* genes, the red line represents the *Arabidopsis* have collinearity of *WD40* genes, the green line represents sugar beet and *Arabidopsis* have collinearity *WD40* genes.

To study the occurrence of the *WD40* genes in sugar beet and its evolutionary relationship among different species, we plotted the collinearity map of the *WD40* genes in sugar beet and *Arabidopsis* (**Figure 3**). The result showed that the *BvWD40* genes had fewer duplication events, only 4 pairs (*BvWD40-34*/*BvWD40-141*, *BvWD40-7*/*BvWD40-72, BvWD40-17*/*BvWD40-90*, *BvWD40-11*/*BvWD40-156*) were found. In contrast, *Arabidopsis* had 28 duplication events. However, 58 pairs of duplication events existed between sugar beet and *Arabidopsis*, more than the two species had on their own.

### Gene ontology analysis of *BvWD40* genes

**Figure 4:**
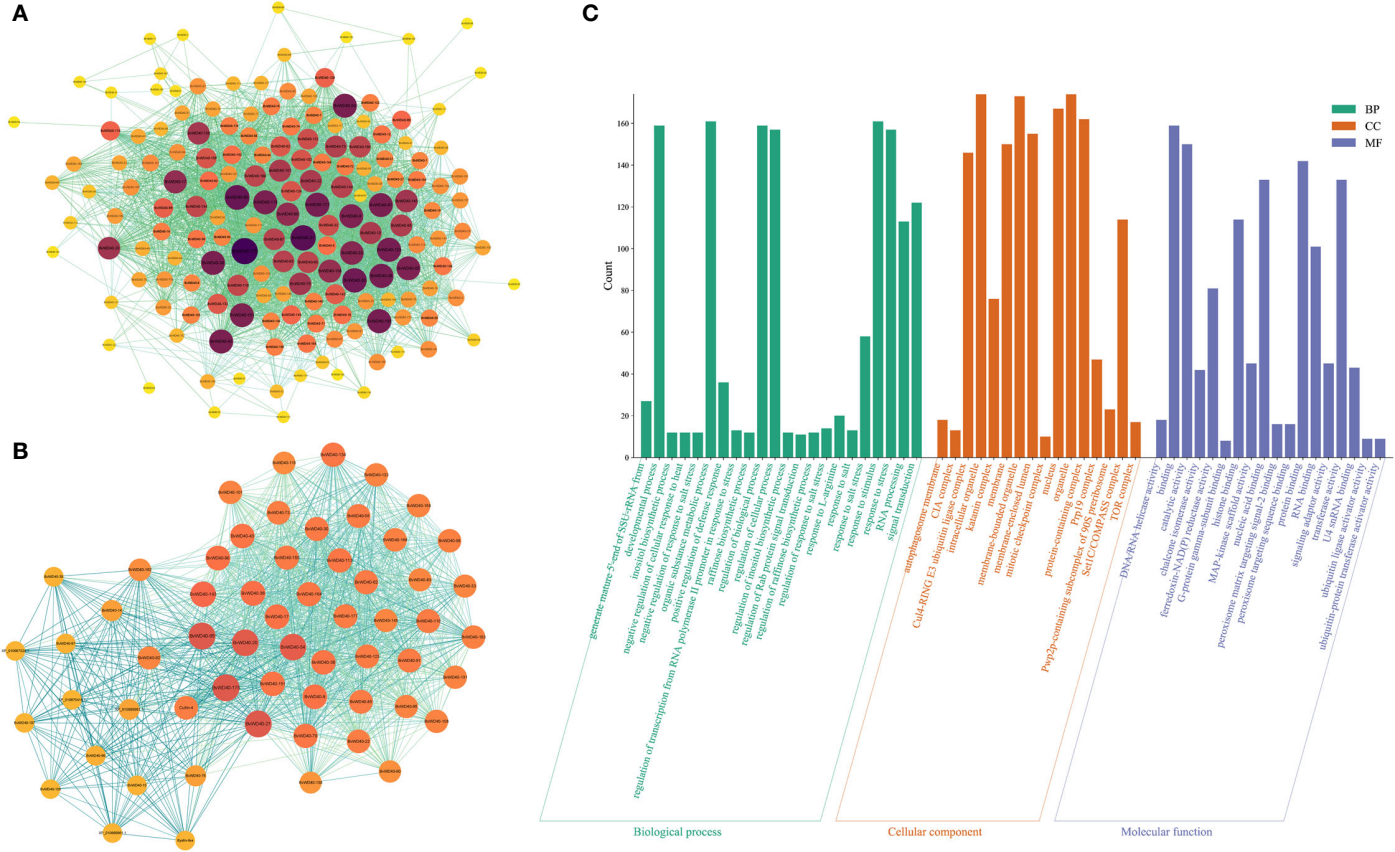
Interaction network of *Bv*WD40 proteins and GO analysis of *BvWD40* genes. **(A)** Interaction network among the *Bv*WD40s. **(B)** Network of interactions between *Bv*WD40s and other proteins. In both images, the sphere size and fill color represent the number of nodes (yellow – dark purple: 1-100), and the line color represents the Combined Score (light green - light blue: 0.4-1) calculated from the String tool. **(C)** *BvWD40* genes ontology analysis, where the abscissa represents the GO item, the ordinate represents the number of genes enriched in GO, with BP, CC and MF representing the biological process, cell composition, and molecular function, respectively.

GO functional enrichment results showed (**Figure 4C**) that a large number of *BvWD40s* were enriched in organ composition, plant development process, regulation of biological processes, catalytic activity, protein binding, signal transduction, nucleotide binding, and other basic biological functions. In addition, a large number of *BvWD40s* were enriched in response to stress such as stimulus-response, heat response, and salt response.

### Analysis of *Bv*WD40 protein interaction network

We obtained the interaction network relationship of the *Bv*WD40 proteins using the String. There were abundant interactions among the *Bv*WD40s (**Figure 4A**). A total of 167 proteins had 3337 interaction relationships, and 50 *Bv*WD40s had more than 50 interaction relationships. *Bv*WD40-175 may interact with 60% of other *Bv*WD40s (100 BvWD40s). It is a homolog of root initiation defect protein 3 (RID3), which was involved in the apical meristem (SAM) regeneration as a negative regulator of the CUC-STM pathway (Tamaki et al., 2009). In order to obtain the interaction network between WD40s and other proteins, we expanded the String network node and found a total of 207 proteins with 4418 interactions. We conducted MCODE analysis on this network and found a key network, which was composed of 53 *Bv*WD40 proteins and 6 non-*Bv*WD40 proteins, with 1160 interactions (**Figure 4B**). In the network, *Bv*WD40-175 and Cullin4 were the *Bv*WD40 and non-WD40 protein with the most interactions (117 and 110 nodes, respectively). Cullin-4 is the scaffold subunit of E3 ligase, which binds to DDB1 and DCAF to play the role of E3 ligase. Some studies have shown that the *Arabidopsis* DCAF protein ABD1 negatively regulates abscisic acid signaling in *Arabidopsis* (Mistry et al., 2020). The tomato DCAF protein DDI1 acts as a substrate receptor for CUL4-DDB1 ubiquitin ligase and actively regulates abiotic stress tolerance in tomato (Miao et al., 2014). It is speculated that *Bv*WD40s may interact with each other to participate in plant development and growth. Most of them may be the substrates of Cullin-4 and may be involved in abiotic stress responses.

### Expression pattern of *BvWD40* genes under salt stress

To further explore the response of *BvWD40* genes under salt stress, we plotted the expression profile of all the *BvWD40s* in cultivated *B. vulgaris* under salt stress (**Supplementary Figure 4**). We studied the expression patterns of *BvWD40s* in two tissues at different times under 200 mM, 300 mM or 400 mM NaCl treatment. Between 12 h and 72 h under 300 mM NaCl treatment, there were a large number of *BvWD40s* changed expression in leaves and roots. The number of genes responding to salt stress in the roots reached the maximum at 72 h (35 genes), and those in the leaf reached the maximum at 24 h (39 genes). We found that six *BvWD40s* in the roots and five *BvWD40s* in the leaves consistently responded to salt stress for 12 to 72 hours. In the leaves of the *BvM14* line, there were most 49 *BvWD40s* responded to 200 mM salt stress and 32 *BvWD40s* responded to both 200 mM and 400 mM salt stress. In the roots, there were most 92 *BvWD40s* responded to 200 mM salt stress and 58 *BvWD40s* changed in response to salt stress at different concentrations. In both tissues, 15 *BvWD40s* (*BvWD40-6, 11, 38, 44, 62, 66, 72, 82, 83, 87, 109, 126, 141, 152*, and *162*) simultaneously responded to different concentrations of salt stress. These results indicate that many *BvWD40s* in different tissues are responsive to salt stress conditions. In different sugar beet lines, there are unique and shared salt-stress responses of the *BvWD40s* at the spatiotemporal level. Clearly, many *BvWD40s* may play important roles in sugar beet salt tolerance, and those unique to the *BvM14* and changed in both leaves and roots may possess high value for biotechnological applications.

### Cloning, tissue specific expression, and subcellular localization of *BvWD40-82*

**Figure 5:**
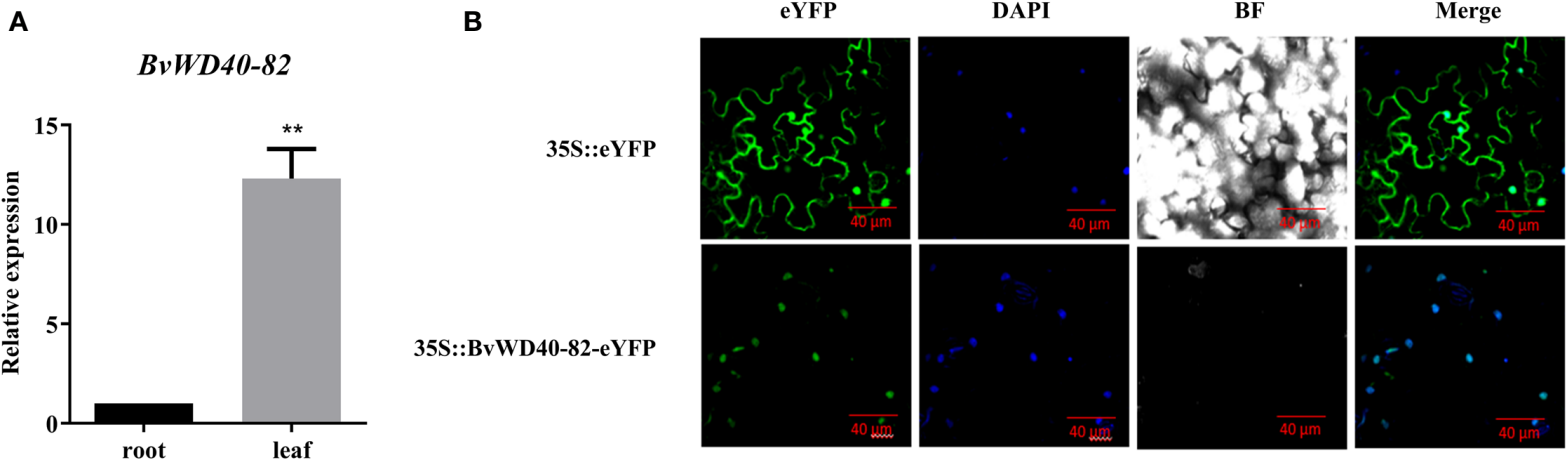
Tissue specificity analysis of *BvWD40-82* gene and subcellular localization of *Bv*WD40-82 protein. **(A)** qRT-PCR analysis of *BvWD40-82* expression levels in different tissues, using *18sRNA* gene as the reference gene, the data are the mean ± SD (n = 3), ** represents the chi-square test at p<0.01 significant difference. **(B)** Subcellular localization of *Bv*WD40-82 protein in tobacco with 35S::eYFP as blank control.

According to the expression analysis of *BvWD40* genes, we found that the *BvWD40-82* gene was up-regulated in both the leaves and roots of the *BvM14* line under the 200 mM and 400 mM NaCl. To the best of our knowledge, no previous studies on the salt tolerance function of the *BvWD40-82* were reported. Here we cloned the *BvWD40-82* gene from the *BvM14* line (**Supplementary Figure 5**). The tissue-specific expression analysis showed that the expression level of the *BvWD40-82* gene in leaves was 12.3-fold higher than in roots (**Figure 5A**). GFP imaging showed that *Bv*WD40-82 protein was localized to the nucleus (**Figure 5B**).

### Generation of *BvWD40-82* CRISPR mutant and overexpression transgenic *Arabidopsis*

For functional characterization, we identified the most homologous gene of *BvWD40-82* in *A. thaliana*, *AtUTP18* (*AT5G14050*). Due to the lack of *AtUTP18* mutants in public repositories, we generated a mutant of *AtUTP18* using a CRISPR/Cas9 method (Pawluk et al., 2016). Sequencing results showed that the *utp18* mutant had a single peak indicative of an inserted thymine at 872 bp of the CDS of the *AtUTP18* gene (only one exon), causing a frameshift mutation and termination at 879 bp (knockout, KO) (**Supplementary Figure 5**). In addition, we have also created *BvWD40-82* overexpression (OE) *Arabidopsis* and complementation line of the *utp18* mutant (CO). Genotyping and RT-PCR results showed that *the BvWD40-82* gene was detected at the DNA group level and RNA level in OE and CO, but not in WT and KO, and *AtUTP18* was knocked out in KO (**Supplementary Figure 5**). The results proved that transgenic plants and the knockout mutant are reliable and can be used for subsequent functional analysis.

### Phenotypic analysis of transgenic plants, WT and KO under salt stress

**Figure 6:**
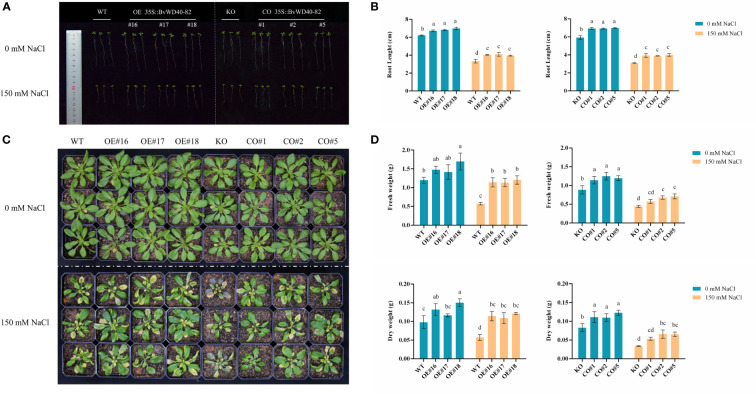
Phenotype analysis of *BvWD40-82* gene in *Arabidopsis* salt tolerance. **(A, B)**, Root length analysis of transgenic *Arabidopsis* seedlings before and after salt treatment. **(C, D)**. Comparison of growth status, fresh weight, and dry weight of transgenic *Arabidopsis* before and after salt treatment. OE represents overexpression lines, CO represents complementation lines, KO represents knockout mutant, and WT represents wild-type plants.

The roots of OE and CO lines were longer than those of WT and KO under both the control and salt treatment conditions. These results indicate that the *BvWD40-82* gene could promote the root growth of plants at the seedling stage, and at the same time, increase the tolerance of plants to salt stress by promoting root growth (**Figures 6A, B**). Surprisingly, the KO and WT exhibited similar root growth under the control and salt stress conditions. This may be attributed to potentially redundant functions of *AtUTP18* homologs in the *Arabidopsis* roots.

In contrast to the early seedling stage of *Arabidopsis*, at the rosette stage the growth parameters (fresh weight (FW) and dry weight (DW)) of the mutant line showed significant differences from the WT and transgenic lines. The leaves of the KO line withered the most, and showed a stress-related black-purple color (**Figure 6C**), while the FW and DW of WT, OE and CO lines were higher than the mutant line under both the control and salt stress conditions (**Figure 6D**). Compared to WT plants, the FW and DW of OE and CO lines were significantly higher (**Figure 6D**). These results showed that *BvWD40-82* may increase the biomass of transgenic lines under salt stress.

### Physiological, biochemical and salt-stress gene expression analyses

**Figure 7:**
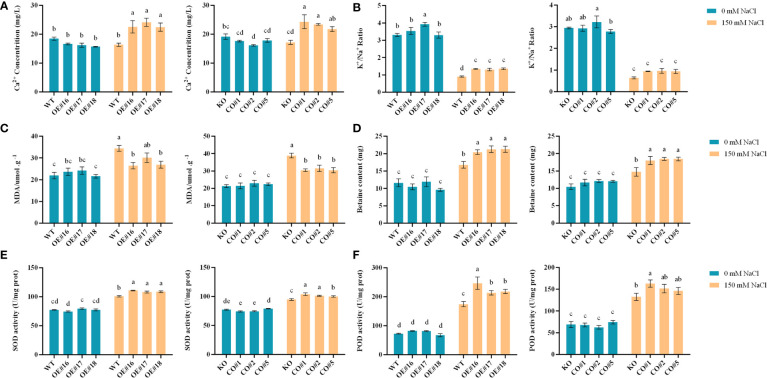
Physiological and biochemical analyses of different plants including OE, CO, KO and WT. **(A-F)**. The Ca^2+^ content, K^+^/Na^+^ content ratio, MDA content, betaine, SOD, and POD enzyme activity of each plant. Lowercase letters indicate significant difference between different groups.

In addition to growth phenotypes, here we profile physiological and biochemical changes, as well as salt-stress responsive pathways in the transgenic, WT and CO plants. There were no significant differences in Ca^2+^ contents and K^+^/Na^+^ content ratios between the transgenic lines (**Figures 7A, B**), WT, and KO lines under control. Under salt stress, the Ca^2+^ contents and K^+^/Na^+^ ratios of the OE lines and CO lines were significantly higher than those of WT and KO lines. Under control, the MDA and betaine contents in the WT and KO did not show significant differences (**Figures 7C, D**). Under salt stress, the MDA contents of the OE and CO lines were significantly lower than those of the WT and KO, and the differences of betaine contents were opposite to those of the MDA in the different plants. Under control, the SOD and POD activities of the transgenic lines, WT and KO lines were not significantly different. Under the salt stress, the SOD and POD activities of OE lines and CO lines were significantly higher than those of WT and KO plants (**Figures 7E, F**).

**Figure 8:**
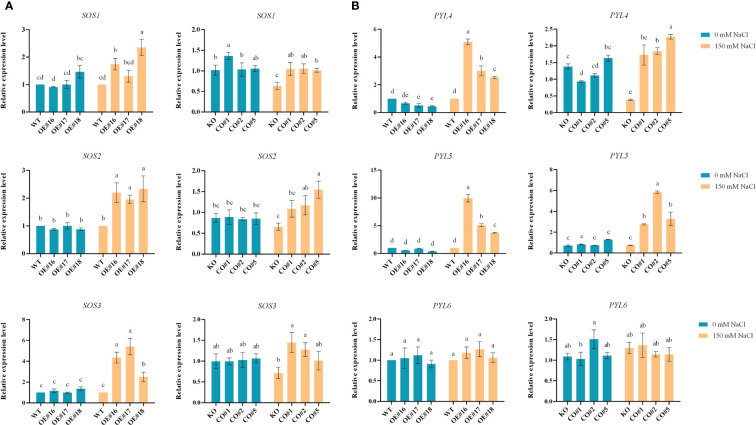
Gene expression analysis of potential *BvWD40-82* mediated salt stress pathways. **(A)** SOS-related pathway expression analysis. **(B)** ABA-related pathway expression analysis. Lowercase letters indicate significant difference between different groups.

To test the potential salt-stress response pathways affected by the *BvWD40-82* gene in plant response to salt stress, we selected the salt-overly-sensitive (SOS) pathway and ABA pathway and measured the expression levels of key genes in the pathways. Under control, the expression levels of relevant genes in each line were similar, while under salt stress the expression levels of SOS pathway-related genes *SOS1*, *SOS2*, and *SOS3* (**Figure 8A**) in OE lines and CO lines were significantly higher than those of the WT and KO lines. With the exception of *PYL6*, the expression levels of *PYL4* and *PYL5* in the ABA pathway-related genes were induced by salt stress, and they were significantly higher in the OE and CO lines than those in the WT and KO (**Figure 8B**).

## Discussion

### Evolution of the WD40 protein family in sugar beet

Sugar beet is an important sugar crop in the world and salt stress can seriously compromise its yield. Therefore, the specific roles of WD40 protein in sugar beet and whether WD40 protein affects its salt tolerance and yield remain to be investigated. In this study, bioinformatics tools and public databases were used to conduct a whole genome analysis of the WD40 protein family in sugar beet. A total of 177 *Bv*WD40s were obtained, and the number was smaller than most plant WD40 protein families, such as rice (Ouyang et al., 2012), cucumber, and *Arabidopsis* (Li et al., 2014). Although their genome sizes are smaller than sugar beet, they have more WD40 proteins. Previous studies have shown that duplication events are the main reason for the large family size (Cannon et al., 2004). Thus, it may be logical to deduce that the sugar beet genome had fewer duplication events than those three species (**Supplementary Figure 6**). The topological structure of the evolutionary tree divided the *Bv*WD40 proteins into 14 groups. The composition of the domains in the same group was similar, indicating that the closely related *Bv*WD40s may have redundant or cooperative functions (Feng et al., 2019). The phylogenetic tree also reflects the differences in physicochemical properties of *Bv*WD40s, which are similar to WD40 proteins in other species (Sun et al., 2020; Chen et al., 2023).

To explore the evolutionary relationship between *WD40* genes, *Arabidopsis WD40* genes and sugar beet *WD40* genes were used to construct a collinearity map. In both sugar beet and *Arabidopsis*, only a small amount of *WD40* genes is produced by duplication events (4 pairs and 28 pairs, respectively), while most *WD* genes did not undergo gene duplication events, indicating that *WD40* is a relatively old gene family. It also indicated that the *WD40* genes were present before the differentiation between sugar beet and *Arabidopsis*, and has a certain diversity due to a series of duplication events after differentiation (Yang et al., 2020). This result is consistent with the results of other studies (Ouyang et al., 2012; Hu et al., 2018).

### The *BvWD40* genes are involved in many biological processes

To further investigate the possible function of *Bv*WD40 proteins in sugar beet, its gene ontology (GO) was annotated. GO annotation and enrichment results indicate that the functions of *BvWD40s* were diverse, including response to salt stress. The enrichment results were similar to those of potato (Tao et al., 2019), barley (Chen et al., 2023), cotton (Salih et al., 2018), and rose (Sun et al., 2020), indicating species conservation. Functions in plant development, ubiquitination, organ composition, microtubule cleavage, signal transduction, protein binding, nucleotide binding, stimulation response, and other biological functions were enriched, indicating the functional diversity of the *WD40* genes. Compared with *WD40* genes of other species, a large number of *BvWD40s* were enriched in heat response, salt response, as well as in raffinose synthesis and inositol synthesis. Accumulation of raffinose was reported to contribute to the salt tolerance of sugar beet (Naguib et al., 2021), and *Populus* inositol transporter gene *PtINT1b* can enhance the salt tolerance of the transgenic plants (Zhang et al., 2023). Thus, *BvWD40s* may have a unique role in salt stress tolerance. Other enriched functions of *BvWD40s* also showed correlation with salt stress, such as peroxisome biogenesis, TOR signal transduction, MAPK complex, Katanin complex, RNA binding, autophagy, and ubiquitination. Previous studies have shown that *OsPEX11*, a peroxisome biogenesis factor in rice, contributes to salt stress tolerance in rice (Cui et al., 2016), TOR signaling is necessary during plant stress (Haq et al., 2022), and MAPK can mediate salt stress signal transduction in plants (Wu et al., 2023). *AtKATANIN1*, which encodes a microtubule cutting protein, regulates microtubule depolymerization in response to salt stress in *Arabidopsis* (Yang et al., 2019). *OsRGG1*, a gene coding for a γ subunit of G protein, promotes salt tolerance in rice by promoting ROS removal (Swain et al., 2017). An RNA binding protein MUG13.4 can interact with *At*AGO2, and the MUG13.4-*At*AGO2 complex plays an important role in the salt tolerance of *Arabidopsis* (Wang et al., 2019). Previous reports have also shown that autophagy plays an important role in plants coping with adversity, and plants adapt to environmental stresses by selective protein degradation through ubiquitination (Xu and Xue, 2019; Raffeiner et al., 2023). The GO results of *Bv*WD40 proteins are exciting, and consistent with other studies (Sun et al., 2020; Chen et al., 2023). They not only show the potential functional diversity of the *Bv*WD40 proteins, but also provide important insight into understanding the roles of the *Bv*WD40 proteins in sugar beet salt tolerance.

### Subfamily classification supporting the role of *Bv*WD40s in salt stress response and tolerance

It has been widely recognized that the protein domains are highly correlative to their functions (Lees et al., 2016). Therefore, studying the domain compositions of the *Bv*WD40 proteins has potential in predicting their functions and generating testable hypotheses. In this study, 177 *Bv*WD40s were divided into 13 subfamilies according to the composition of their domains. It was noted that subfamily C possesses typical ubiquitination domains such as UBOX, FBOX and RING, indicating involvement in ubiquitination. Previous studies have shown that a soybean *Gm*PUB21 containing UBOX domain negatively regulates drought and salt stress tolerance in soybean (Yang et al., 2022). The RING zinc finger protein with RING domain as an E3 ligase plays an important role in plant growth and abiotic stress tolerance (Han et al., 2022). In addition, *At*SDR containing FBOX domain is involved in abiotic stress in *Arabidopsis* (Li et al., 2022a). Ten *Bv*WD40s were found to contain ubiquitination domains in sugar beet. We hypothesized that these *Bv*WD40s may play a role in abiotic stress tolerance through the activities of proteasomes. It was also noted that subfamily K has only one member, annotated as cellulose synthesis based on its domain function. Plants under salt stress may cope with the damage caused by salt stress by regulating the synthesis and deposition of cell wall (Dabravolski and Isayenkov, 2023). In addition, subfamily A has the largest number of *Bv*WD40s, and previous reports have confirmed that the WD40 protein containing only the WD40 domain positively responds to salt stress in plants. For example, mango *MiTTG1* coding WD40 protein plays an important role in promoting the development of root length and root hair, and the transgenic line has a stronger ability to adapt to salt and drought stresses (Tan et al., 2021). An *LbTTG1* of *L. bicolor* can promote the growth of *Arabidopsis* trichome and actively exudate salt through salt glands to enhance the plant tolerance to salt stress (Yuan et al., 2019). Overexpression of *TaWD40D* could increase the expression of genes related to the SOS pathway in transgenic plants under salt stress, thus enhancing the tolerance of wheat to salt stress (Kong et al., 2015), and *GbLWD1-like* gene in *Ginkgo biloba* can improve the growth of transgenic poplar and increase the expression of salt-stress-related transcription factors, thus improving the salt tolerance of transgenic poplar (Xin et al., 2021). Overexpression of another *OsABT* gene improved rice salt stress tolerance through preventing excessive ROS accumulation, increasing intracellular K^+^/Na^+^, decreasing ABA synthesis, and activating ABA responsive gene expression and ABA signaling pathway (Wen et al., 2022).The WD40 protein REBC in quinoa is involved in the formation of epidermal bladder cells, and mutation of *REBC* led to salt stress sensitivity (Imamura et al., 2020). Different subfamily members with different domains may participate in multiple biological functions. Studies on some domains indicate that they may also be associated with salt stress, while studies on members of subfamily A indicate that this subfamily has a higher correlation with salt stress than other subfamilies. These results support that *Bv*WD40 proteins may play a key role in plant salt stress tolerance.

### *BvWD40-82* enhanced salt tolerance in transgenic *Arabidopsis*

*BvM14* line is an excellent germplasm resource independently created in Profession Li’s laboratory. It grows normally under high salt concentration, while the cultivated sugar beet cannot. At present, some excellent salt tolerance genes have been isolated from the *BvM14* line, and they are considered to be important biomarkers for salt tolerance of sugar beet (Ma et al., 2017; Ji et al., 2019). However, no *WD40-related* genes have been characterized to be related to the salt tolerance of sugar beet. First, expression analysis of the *BvWD40s* under salt stress showed that a large number of *BvWD40s* responded to different concentrations of salt stress in different tissues of sugar beet, and *BvWD40-82* had a significant response. Second, the *BvWD40-82* contains only the WD40 domain, and belong to subfamily A, indicating that the *BvWD40-82* may be involved in plant salt tolerance. Therefore, *BvWD40-82* was cloned for functional studies using reverse genetics in *Arabidopsis*. Under salt stress, MDA can be increased significantly under salt stress, thus damaging the cell membranes (Liu et al., 2023a). The *BvWD40-82* gene can promote root growth of the *BvWD40-82* transgenic plants, reduce the accumulation of MDA under salt stress, thereby reducing the plant damage under salt stress, and improve the accumulation of betaine in transgenic plants to maintain the osmotic pressure of plant cells (Annunziata et al., 2019). Third, heterologous overexpression and complementation of the *BvWD40-82* gene promoted Na^+^ efflux and inhibited K^+^ efflux, maintained a high K^+^/Na^+^ ratio, increase Ca^2+^ content, and improved plant salt tolerance (Zhao et al., 2021; Liu et al., 2023b). Fourth, *BvWD40-82* positively regulated SOD and POD activities under salt stress, thus maintaining the ROS homeostasis in plants (Yang and Guo, 2018). Additionally, under salt stress, the *BvWD40-82* enhanced salt tolerance through regulating the SOS signal pathways related-gene expression, activated the Na^+^/H^+^ transport channels and K^+^ intake (Xu et al., 2023), and increased the expression of ABA receptor genes (*PYL4*, *PYL5*) in the ABA signaling pathway (Wen et al., 2022). Functional characterization of *BvWD40-82* clearly showed that the *BvWD40-82* played an important role in plant response to salt stress. It can improve the salt tolerance of *Arabidopsis*. However, further investigation is needed to elucidate the molecular mechanisms underlying the *BvWD40-82* function in plant salt tolerance.

## Conclusion

This study identified 177 *Bv*WD40 proteins from the sugar beet genome and described their gene and protein structures, chromosome distribution, and evolutionary characteristics. The response of *BvWD40s* to salt stress was characterized and a potential salt tolerance gene *BvWD40-82* was isolated. The functions of the *BvWD40-82* gene in salt tolerance were studied through a series of molecular, physiological and biochemical analyses. The *BvWD40-82* gene can improve the salt tolerance of transgenic plants by increasing the contents of osmolytes and antioxidant enzyme activities, maintaining intracellular ion homeostasis and increasing the expression of genes related to SOS and ABA pathways. This study revealed the important role of *BvWD40s* in the response to salt stress in sugar beet and provided a theoretical basis for improving sugar beet tolerance to salt stress, and the *BvWD40s* may also serve as important resources for the genetic breeding of other crops.

## Data availability statement

The datasets presented in this study can be found in online repositories. The names of the repository/repositories and accession number(s) can be found in the article/**Supplementary Material**.

## Author contributions

Writing and editing: ZW and SC. Molecular experiment and assistance in editing: TZ and JL. Idea conception and supervision: BY and HL. Document check: IG and DZ. Conception and modification: SC and HL. All authors have read and agreed to the published version of the manuscript.
