# Supplementary material for: Genome-wide analysis of WD40 protein family and functional characterization of BvWD40-82 in sugar beet
Source: Front Plant Sci. 2023 Jun 2;14:1185440. doi: 10.3389/fpls.2023.1185440 (PMC10272600; doi:10.3389/fpls.2023.1185440)
Supplement: Supplementary file 1 [file DataSheet_1.zip › Supplementary Material/Supplementary figures.docx]

Supplementary Material

Genome-wide analysis of WD40 protein family and functional study of *BvWD40-82* gene in sugar beet (*Beta vulgaris* L.)

**Zhirui Wu, Tingyue Zhang, Jinna Li, Sixue Chen, Bing Yu* and Haiying Li***

*** Correspondence:** Bing Yu: ybgirl1234@sina.com; Haiying Li: lvzh3000@sina.com

**Supplementary Figure**





**Supplementary Figure 1.** Phylogenetic tree binding conserved motif and domain map of 177 BvWD40s.


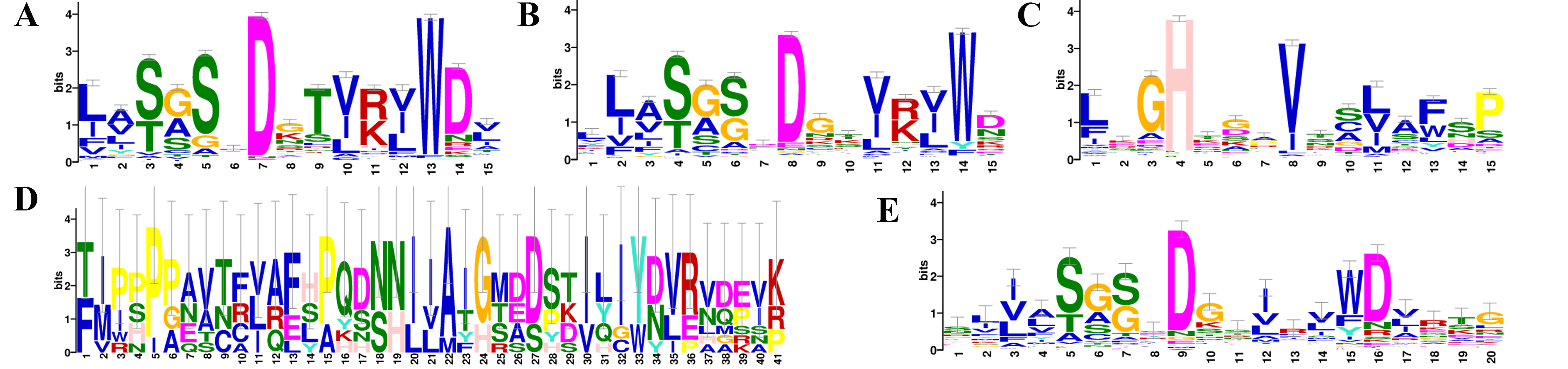


**Supplementary Figure 2.** The graph of conserved motifs downloaded in MEME. (**A**). Motif 1. (**B**). Motif 2. (**C**). Motif 4. (**D**). Motif 16. (**E**). Motif 18.


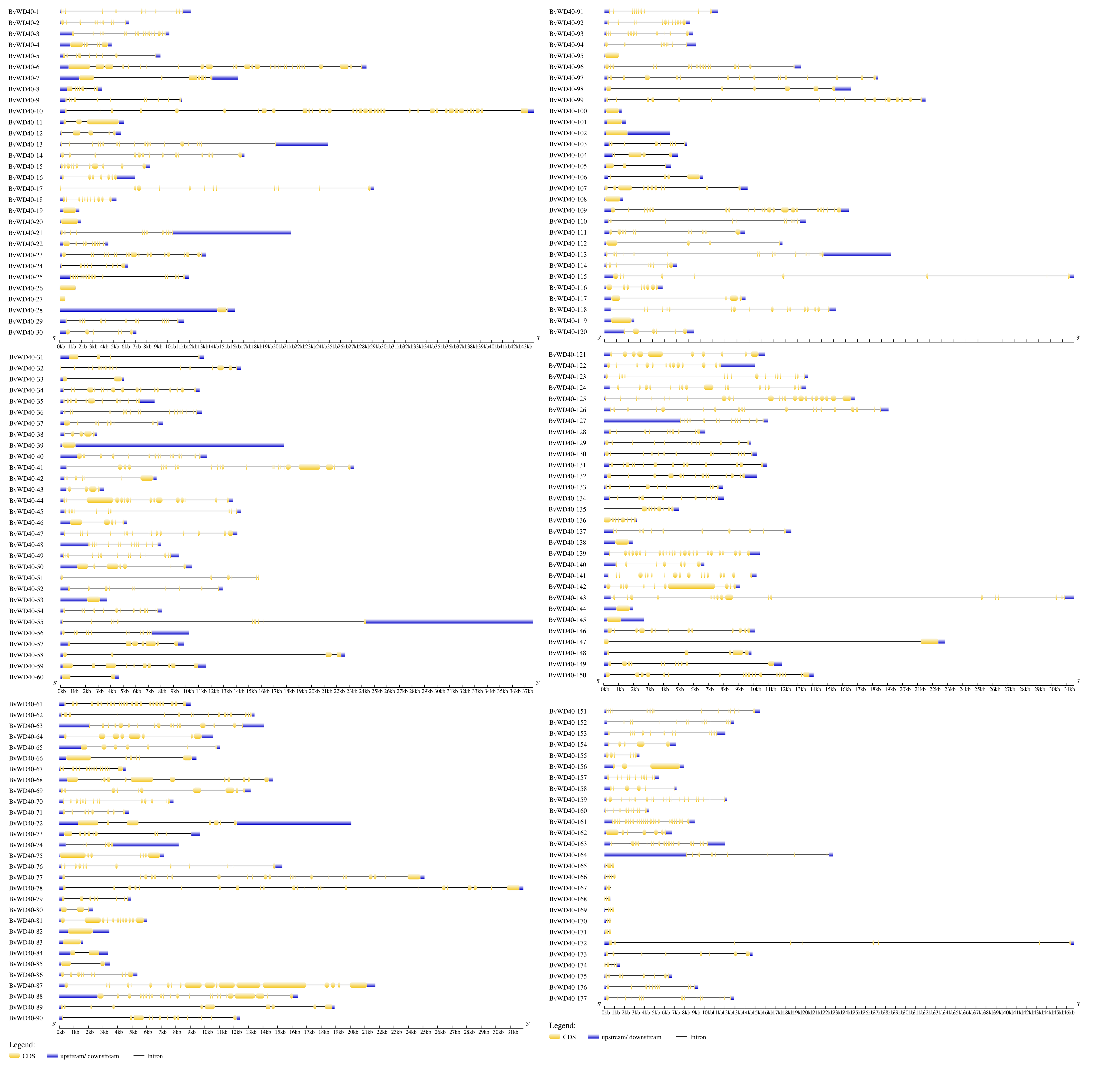


**Supplementary Figure 3.** Gene structure maps of *BvWD40s*. Drawn by GSDS.





**Supplementary Figure 4.** Expression profile analysis of *BvWD40* genes under salt stress. The filling color of each cell indicates the expression pattern of the gene under specific treatment. The expression pattern of *BvWD40s* is reflected by the logarithm of the expression ratio under different treatments, with red representing up-regulation and blue representing down-regulation. Treatments 1-4 indicate the gene expression patterns in leaves of *B. Vulgaris* at 12, 24, 48, and 72 h under 300 mM NaCl stress, and CK1 was the control group. Treatments 5-8 indicate the expression pattern in *B. Vulgaris* roots at 12, 24, 48, and 72 hours under 300 mM NaCl stress, and CK2 was the control group. Treatments 9-10 represent the gene expression patterns in the leaves of the *BvM14* line under 200 mM and 400 mM NaCl stress, and CK3 was the control group. Treatments11-12 represent the gene expression patterns in the roots of the *BvM14* line under 200 mM and 400 mM NaCl stress, and CK4 was the control group.


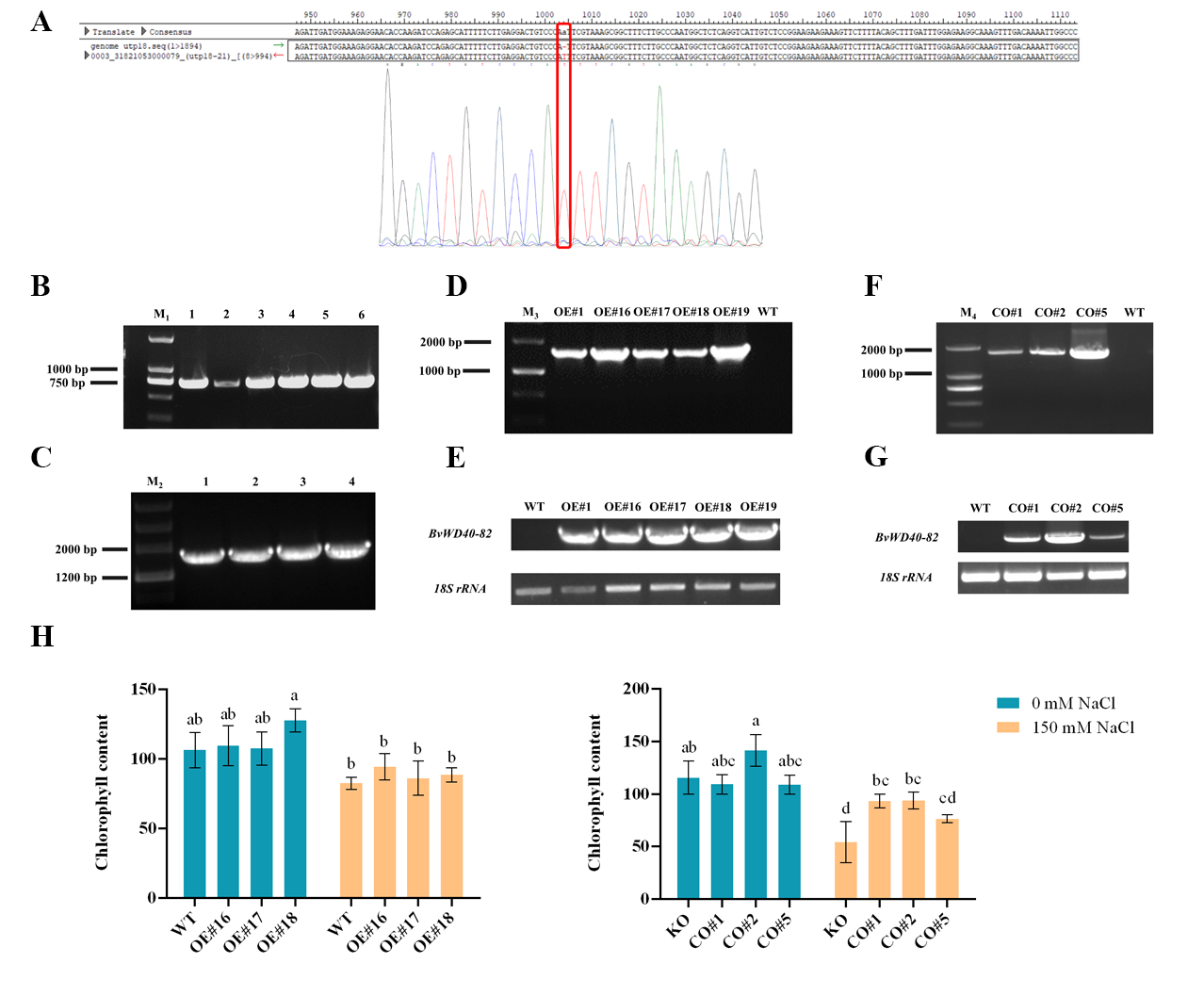


**Supplementary Figure 5.** The cloning of *BvWD40-82*, and the generation of mutation and transgenic lines, and the chlorophyl content of wild and transgenic plant under salt stress. (**A**). The sequencing of *utp18* mutant, the red box is the mutation site. The figures in the picture do not represent the full length of CDS sequence of *AtUTP18*, but contain an additional part of the sequencing result. (**B**). The construction of mutant vector, M_1_: DL 2000 DNA Marker, 1-6: the appropriate target is constructed onto the pNGG2F vector, the length of the target product was correctly 750 bp. (**C**). The cloning of *BvWD40-82*, M_2_: DNA Marker Ⅲ, 1-5: the length of the *BvWD40-82* was correctly 1800 bp. (**D-E**) Heterologous overexpression (OE#16, OE17, OE18) and heterologous complementation (CO#1, CO#2, CO#5) have a detected bands of *BvWD40-82* at the DNA level, control (WT) has not detected, M_3_, M_4_: DL 2000 DNA Maker. **(F-G)**. OE lines and CO lines have a detected bands of *BvWD40-82* at the RNA level, and control has not detected, *18s* RNA as reference gene. (**H**) The chlorophyl content of -type and transgenic plant under salt stress. OE represents overexpression lines, CO represents complementation lines, KO represents knockout mutant, and WT represents wild-type plants.


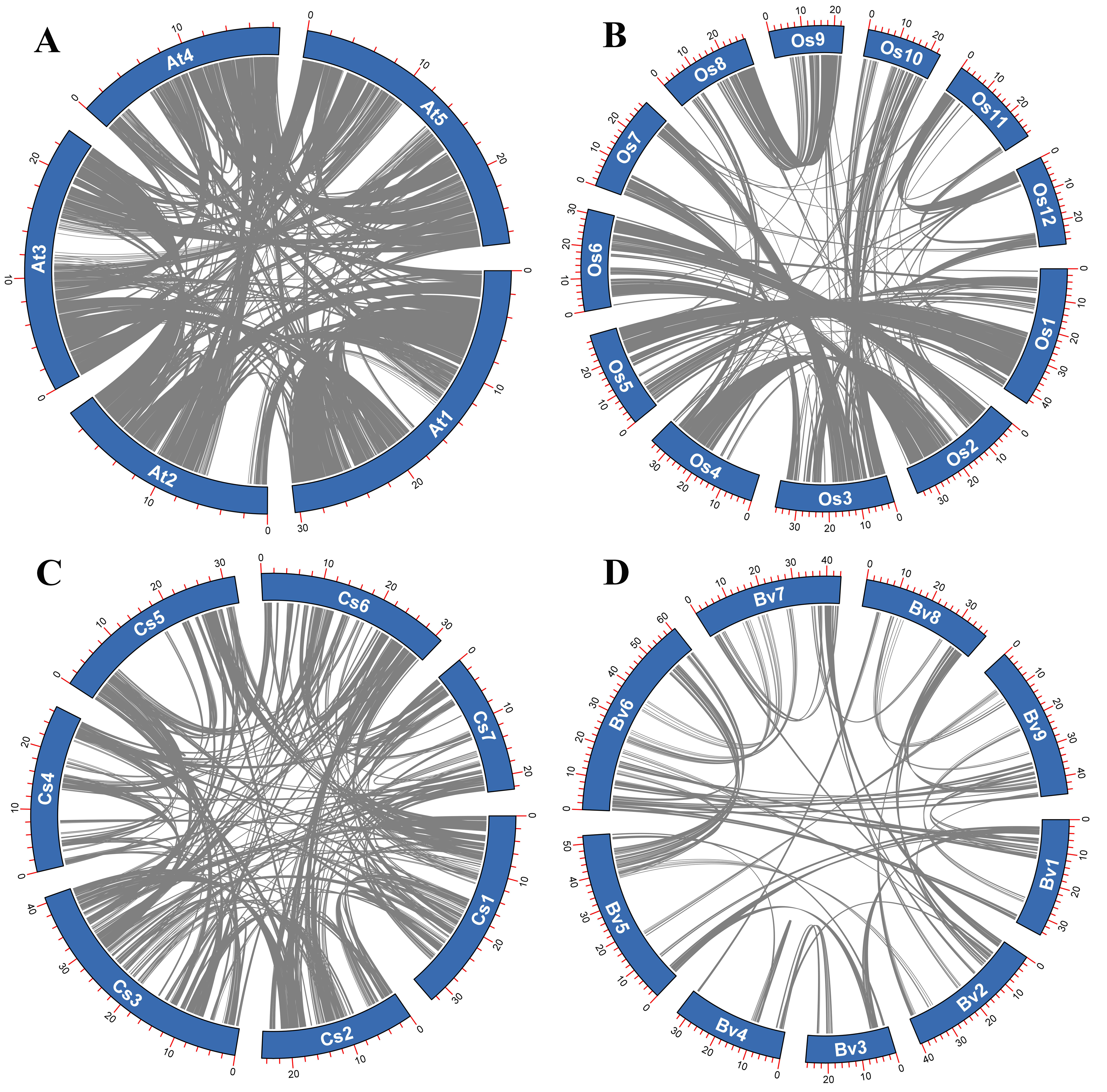


**Supplementary Figure 6.** Collinearity maps of the genomes of rice, cucumber, sugar beet and *Arabidopsis thaliana*. **(A)**. *Arabidopsis thaliana*. **(B).** rice. **(C).** cucumber. **(D).** sugar beet.
